# Uncovering the true features of dystrophin gene rearrangement and improving the molecular diagnosis of Duchenne and Becker muscular dystrophies

**DOI:** 10.1016/j.isci.2023.108365

**Published:** 2023-10-30

**Authors:** Chao Ling, Yi Dai, Chang Geng, Shirang Pan, Weipeng Quan, Qingyun Ding, Xunzhe Yang, Dongchao Shen, Qing Tao, Jingjing Li, Jia Li, Yinbing Wang, Shan Jiang, Yang Wang, Lin Chen, Liying Cui, Depeng Wang

**Affiliations:** 1 The Laboratory of Clinical Genetics, Medical Research Center, Peking Union Medical College Hospital, Chinese Academy of Medical Science & Peking Union Medical College, Beijing 100730, China; 2 Department of Neurology, Peking Union Medical College Hospital, Chinese Academy of Medical Science & Peking Union Medical College, Beijing 100730, China; 3 State Key Laboratory of Complex Severe and Rare Diseases, Peking Union Medical College Hospital, Chinese Academy of Medical Science & Peking Union Medical College, Beijing 100730, China; 4 Grandomics Biosciences, Beijing 102200, China

**Keywords:** Health sciences, Clinical genetics, Pediatrics

## Abstract

Duchenne and Becker muscular dystrophies (DMD/BMD) are caused by complex mutations in the dystrophin gene (*DMD*). Currently, there is no integrative method for the precise detection of all potential *DMD* variants, a gap which we aimed to address using long-read sequencing. The captured long-read sequencing panel developed in this study was applied to 129 subjects, including 11 who had previously unsolved cases. The results showed that this method accurately detected *DMD* mutations, ranging from single-nucleotide variations to structural variations. Furthermore, our findings revealed that continuous exon duplication/deletion in the DMD/BMD cohort may be attributed to complex segmental rearrangements and that noncontiguous duplication/deletion is generally attributed to intragenic inversion or interchromosome translocation. Mutations in the deep introns were confirmed to produce a pseudoexon. Moreover, variations in female carriers were precisely identified. The integrated and precise *DMD* gene screening method proposed in this study could improve the molecular diagnosis of DMD/BMD.

## Highlights

• Long-read sequencing can detect *DMD* mutations that involve both SNVs and complex SVs
• Non-contiguous changes due to intragenic inversion or interchromosome translocation
• Deep intron mutations generate pseudoexons, advancing DMD/BMD genetics
• The findings could enhance diagnoses and inform clinicians and researchers

## Graphical abstract

**Figure:**
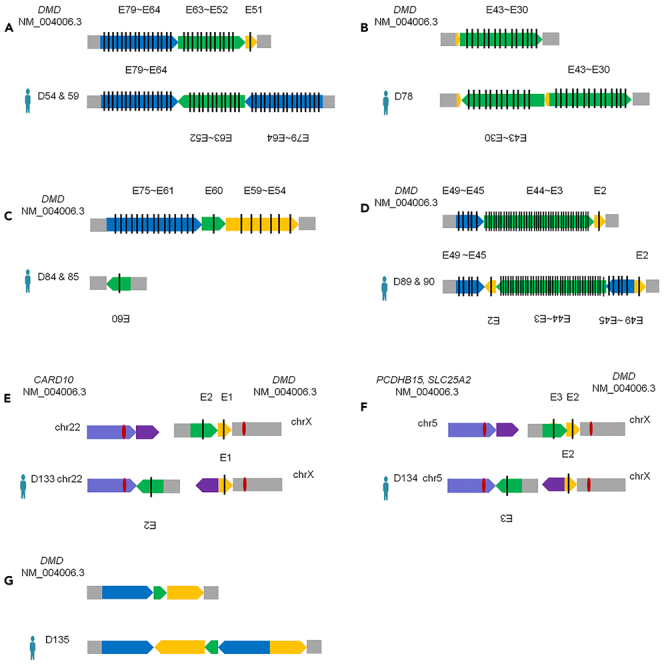

## Introduction

Molecular genetic studies have indicated that both Duchenne (OMIM: 310200) muscular dystrophy (DMD) and Becker (OMIM: 300376) muscular dystrophy (BMD) are caused by mutations in the dystrophin gene (*DMD*).^1^ In male probands, hemizygous pathogenic variants are frequently identified in *DMD*. In contrast, females are usually asymptomatic carriers; however, some female carriers show mild skeletal muscle and/or cardiac symptoms of DMD/BMD and are referred to as manifesting carriers. In patients with DMD/BMD, 68–70% of mutations are single or multiple exon deletions, and 8–11% are duplications of the *DMD* gene. Additionally, 18–20% were small mutations, including insertions, deletions, and single-nucleotide substitutions in exons and introns.^2^^,^^3^^,^^4^ In the case of patients with DMD/BMD, diverse methods for causative mutation investigation have been employed, of which multiplex ligation-dependent probe amplification (MLPA) is commonly and preferentially applied to simultaneously screen all 79 exons of *DMD* for deletions/duplications.^5^ However, single exon deletion still requires additional validation owing to the probe binding problem. Moreover, MLPA is not suitable for detecting single nucleotide variants (SNVs) and small indels.^6^ High-throughput sequencing (short-read sequencing) technologies are widely applied to call SNVs, microdeletions, and microduplications. Depending on the read count, breakpoints for most of the deletions can be identified; however, the generation of accurate results for duplications or complex structural variants is difficult owing to the limitation of read length (100–300 bp).^7^^,^^8^^,^^9^ Nanochannel-based next-generation mapping enables the sensitive detection of pathogenic structural variations (SVs), which can be missed by PCR-based techniques or chromosomal microarrays; however, the resolution of breakpoints is limited to endonuclease nicking site density.^10^ Approximately 2–7% of patients with DMD/BMD cannot be diagnosed using MLPA or high-throughput sequencing/mapping methods, which may be attributed to deep intronic variations, including microindels, substitutions, and large-scale deletions and duplications.^3^^,^^11^^,^^12^^,^^13^ RNA studies have shown that deep intronic mutations can create new splicing sites and generate pseudoexons.^13^^,^^14^^,^^15^ Thus, RNA analysis of muscle tissue using RT-PCR or short-read sequencing could serve as a genetic diagnostic tool for these rare mutations; however, this method requires a highly invasive muscle biopsy procedure. Moreover, clinicians usually require further investigations of DNA mutations for gene therapy and prenatal diagnosis. Therefore, we believe that a method that can obtain an accurate and full spectrum of mutation types of the *DMD* gene based on genomic DNA resources could benefit the molecular diagnosis and treatment of patients with DMD/BMD.

Long-read sequencing, represented by two mainstream platforms: Oxford Nanopore Technologies (ONT) and Pacific Biosciences (PB) single-molecule real-time sequencing, continues to rapidly improve throughput and reduce costs.^16^^,^^17^ Compared to conventional methods, long-read sequencing technologies offer more possibilities, especially in the detection of SVs and complex rearrangements, which involve duplications,^18^^,^^19^ whereas SNVs and small indels can also be accurately identified with adequate read depth (>30×) and HiFi sequencing technology.^20^ Additionally, targeted long-read sequencing is considered to be an efficient and cost-effective method for complex variant detection.^21^ Therefore, long-read sequencing can be used as a unified methodology to detect all heterogeneous sets of pathogenic mutations in the *DMD* gene. Accordingly, we designed a complete *DMD* gene panel based on long-read sequencing technology. Furthermore, to uncover the true features of *DMD* mutations, we performed genetic sequencing and analyzed 129 patients with DMD/BMD using ONT and PB platforms.

## Results

### Patient information

**Figure 1:**
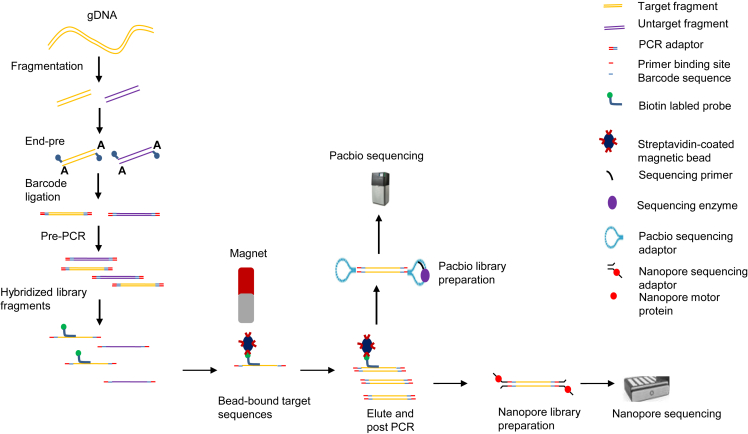
Flow chart of captured long-read sequencing The capture and library preparation of the entire genomic *DMD* gene are shown step-by-step for both PB and ONT platforms, respectively.

Among the 129 subjects included in this study, there were 76 males diagnosed with DMD, 16 males with BMD, seven males with intermediate muscular dystrophy (IMD), and two symptomatic female carriers. Additionally, 24 asymptomatic female carriers and four normal subjects (one male and three females) were included. Of the subjects, 76 were identified by the MLPA method, 33 had mutations that could be detected by conventional short-read sequencing methods, such as whole exome sequencing (WES) or *DMD* gene panel, and 11 were specifically sampled for this study due to the failure of genetic diagnosis using MPLA or NGS-based methods. Additionally, nine relatives who had not been previously diagnosed using MLPA or short-read sequencing were enrolled. In this cohort, 70 subjects were sequenced using the ONT platform, 51 using the PB platform, and eight using both the ONT and PB platforms for parallel validation (Figure 1; Tables S1 and S2).

### Sequencing data overview

A total of 137 independent long-read datasets were generated for the 129 subjects. An average of 118,433 and 1,261,799 reads were collected from the PB and ONT datasets, respectively. The PB platform had an average read length of 2,502 bp with a coverage of 96.92% of the target region, while the ONT platform had an average read length of 1,945 bp with a coverage of 99.99% of the target region. The average sequencing depth for PB and ONT was 168 × and 382 ×, respectively.

### Captured long-read sequencing provided a more efficient method for Duchenne muscular dystrophies gene mutation detection

In this study, accurate variations of the *DMD* gene were identified and corrected using the designed long-read sequencing panel, including mutations that had previously failed to be detected. Captured long-read sequencing provides an advantage for large-scale inversion and translocation detection, with SV fragment sizes ranging from 11 kb to 40 Mb. The results showed that translocations, duplications, and inversions had a wider range of alterations than deletions in the *DMD* gene (Tables 1 and 2). In addition, targeted long-read sequencing is capable of detecting microindels and SNVs, including deep intron variations, which is more accurate than short-read sequencing at the same 30× sequencing depth. In this study, 41 patients had SNVs and small indels within the *DMD* gene. Of these, five were deep intron mutations predicted to affect transcription by generating splicing sites and pseudoexons. Moreover, transcriptional alteration in subject D116, who had an ∼11-kb deletion in intron 48 (chrX: 31881062–31892406), was confirmed to generate a 161-bp transcription by muscle biopsy RNA-Seq (Figure S1). All SNV mutations were correctly identified using long-read sequencing platforms. Moreover, the software and analytic process mentioned in the STAR methods section accurately detected SNVs located within the homologous sequence (subject D130) in both the ONT and PB datasets, even in areas where short-read WGS failed. Nonsense and splice-site mutations were more frequently detected in SNVs (Table 1).

**Table 1:** Long-read sequencing allows for efficient *DMD* gene mutation detection

MP	Total count	MF(%)	Detection rate (a^∗^/b^#^)			SV size (bp)
			MLPA	NGS-panel^α^/WESβ/WGSγ	LRSP	
L-Del	34	26.4	33/34	NA	34/34	11,345–652,383
L-Dup	32	25.6	32/32	NA	32/32	12,735–1,260,562
Inversion	9	7.2	0/8	0/1 ^α^	9/9	85,898–1,298,407
Translocation	2	1.6	0/1	0/2 ^1α/1^β	2/2	13,394,796–40,251,284
LDD	7	5.6	4/4	NA	7/7	30,576–8,653,099
Micro-indel	11	8.8	0/9	9/9 ^α^	11/11	1–4
Stop codon	15	12.0	0/15	15/15 ^α^	15/15	0
Missense	2	1.6	0/2	2/2 ^α^	2/2	0
Splicing	8	6.4	0/8	8/8 ^α^	8/8	0
Deep-intron	5	4.0	0/5	0/5 ^4^β/1γ	5/5	0

MP, mutation patterns; L-Del, large sequential deletion; L-Dup, large sequential duplication; LDD, large discontinuous deletion and duplication; MF, mutation frequency; a∗, number of positive detections; b^#^, total number of samples in the detection method; α, NGS-panel; β, WES; γ, WGS; NA, the detection method is NOT used for this pattern; LRSP, long-read sequencing panel; SV, structural variation.

**Table 2:** Captured long-read sequencing corrected MLPA and short-read sequencing results

ID	MLPA (NM_004006.3)	WES/NGS-panel	Long-read sequencing panel (NC_000023.10)
D54/D59	E51del; E64-79dup	NA	g.30866278_31256392dup; g.30866278_31766900inv; g.31766901_31804205del
D78	E30-43dup	NA	g.32235128_32266641dup; g.32235128_32453034inv; g.32266659_32453034dup
D84/D85	E54-59del; E61-75del	NA	g.311648843_31396554del; g.31396556-31482449inv; g.31482452_31692080del
D89/D90	E2dup; E45-49dup	NA	g.31849725_32161440dup; g.31849725_33148131inv; g.32904520_33148131dup
D133	NA	None	NC_000022.10:g.1_37909829delins[NC_000023.10:g.1_33085040] NC_000023.10:g.1_33085051delins[NC_000022.10:g.1_37909760]
D134	NA	None	NC_000005.9:g.1_140663979delins[NC_000023.10:g.1_32953142] NC_000023.10:g.1_32953139delins[NC_000005.9:g.1_140663975]
D135	NA	None	g.33077035_33127081dup; g.33077033_33166191inv; g.33128480_33166191dup

NA, the detection method is NOT used for the subject; SV, structural variation; None, no variant was detected; inv, invention; delins, the HGVS recommendation for the nomenclature of translocation.

### Captured long-read sequencing revealed the precise characteristics of structural variation ligation in Duchenne muscular dystrophies

**Figure 2:**
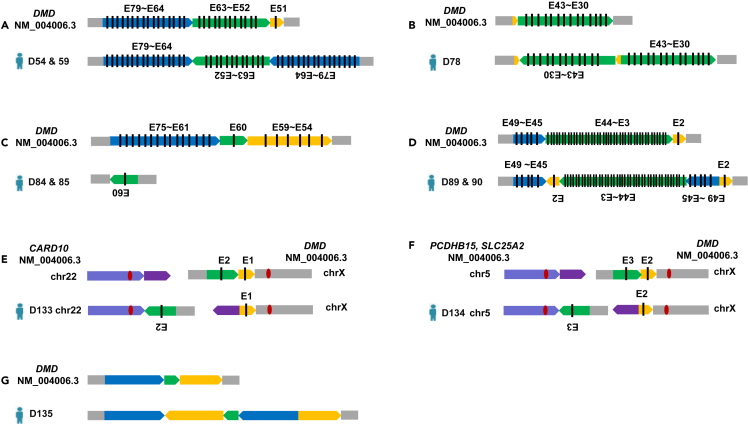
Captured long-read sequencing panel precisely uncovered the SVs of *DMD* (A–G) Long-read sequencing revealed that the non-contiguous variants and the minority of duplications detected by MLPA methods are definitively attributed to inversion (A–D); translocation between autosomal and X chromosomes involving the *DMD* gene is shown (E and F); an inversion occurring in the intron region is indicated (G).

**Figure 3:**
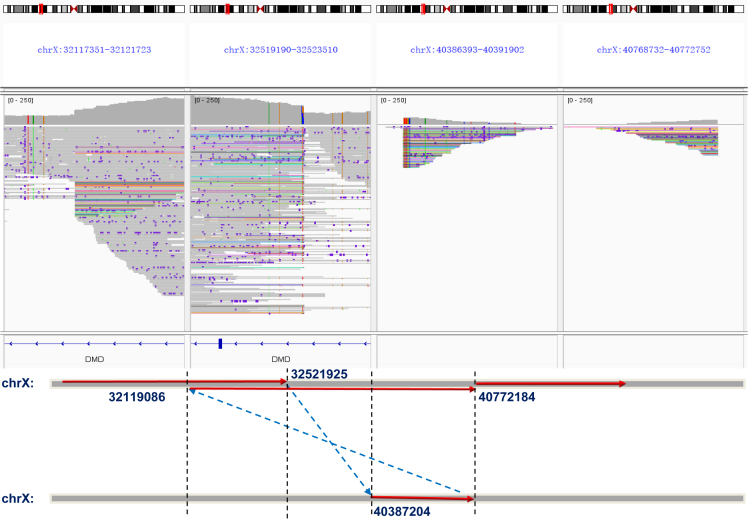
Rare continuous duplication results from deletion and duplication The same colored reads represent the same junction reads, which cross two separate regions of *DMD*.

Using the target long-read sequencing method, a total of 84 patients were found with duplications and/or deletions in the *DMD* gene, of which 32 had duplications, 34 had deletions, nine had inversions, two had translocations, and seven had large discontinuous deletions and duplications (Table S2). Based on the captured long-read sequencing results, we confirmed that all breakpoints of the SVs fell within the intron region and that most contained microinsertions. Additionally, a few subjects had a large fragment (600–1000 bp) insertion between the breakpoints (Table S3); however, sequential ligation was not affected. Moreover, although most of the subjects were found to have head-to-tail ligation, we found a rare subject (D127) with continuous exon 19 to exon 44 duplications that resulted from an upstream to exon 18 deletion (NC_000023.10:g.32521926_40387205del) and an upstream to exon 44 duplication (NC_000023.10:g.32119087_40772185dup). Meanwhile, a nonsense mutation (NM_004006.3:c.5404C>T) was identified. Therefore, the above findings showed that not all sequential duplications were as simple as they seemed in the MLPA results (Figure 2). In addition, in nine of the subjects exhibiting *DMD* intragenic inversion, seven had previously been detected as having discontinuous deletions or duplications using the MLPA method (Figure 3; Table 2). The junction reads produced by inversion and translocation were verified with Sanger sequencing (Figure S2). The captured long-read sequencing panel revealed the characteristics of SV ligation in *DMD*.

### Captured long-read sequencing exhibited higher accuracy for structural variation haplotype detection in female carriers

For SV haplotype detection, samples from 29 female participants were analyzed. We found that the SV haplotypes could be identified by long-read sequencing and were independent of sex. In addition, translocation occurred in two female patients (Figure 3; Table 2; Table S1). Thus, compared with MLPA and short-read sequencing methods, this one-step captured long-read sequencing method could attain higher accuracy in screening female carriers (Tables S1 and S2).

## Discussion

In this study, we investigated the efficacy of a captured long-read sequencing panel and validated its performance in a cohort of 129 individuals. The results demonstrated that, compared with MLPA and short-read sequencing, the captured long-read sequencing method has significant advantages in uncovering the underlying large-scale rearrangements of *DMD*, especially in the detection of deep intron SVs, inversions, and translocations. In addition, this designed panel could capture the homologous region of *DMD*, which the short-read sequencing method could not achieve. Furthermore, at a 30× sequencing depth, this long-read sequencing panel precisely detected microindel mutations and deep intronic SNVs. We also observed that this method provided a more precise identification of SV breakpoint positions in the *DMD* gene. Both the ONT and PB sequencing methods were able to determine the exact breakpoints for deletions, duplications, and other complex rearrangements; however, PB showed slightly better consensus calling than ONT due to a more congruent sequence error pattern. Thus, for SNV detection, PB is more accurate than ONT. However, the accuracy of ONT could be improved by increasing the sequencing depth. Thus, ONT sequencing has the potential to meet the requirements for *DMD* mutation detection.

Although previous studies have characterized the deletion and duplication patterns of *DMD*, accurately identifying the precise junctions of breakpoint sequences, especially those involving duplicates, remains challenging. This highlights the need for further analyses of duplicates to achieve an accurate diagnosis and facilitate antisense-mediated exon-skipping therapy.^22^^,^^23^ Here, long-read sequencing demonstrated robust proficiency in decoding the entire sequence of the *DMD* rearrangement region, surpassing conventional methodologies. In particular, we found that not all duplications were tandem repeats, which directly revealed the nature of exon duplication in *DMD*.

MLPA is an effective method for detecting exon rearrangements in patients with DMD or BMD. These variations include the concurrence of exon deletion and duplication as well as the non-contiguous deletion or duplication of *DMD* exons. However, few studies have provided insight into the underlying sequence of complex SVs in *DMD*.^24^^,^^25^^,^^26^ Our long-read sequencing study suggests that complex SVs are mostly induced by inversion or translocation in unstable regions. Previous studies using chromosomal inversion and translocation detection methods have shown that an inverted duplication occurring next to a terminal deletion is a common rearrangement pattern in cancer and constitutional genomes.^27^^,^^28^ Our junction sequencing analysis revealed that non-contiguous duplications were interconnected with duplications in either the direct or inverted orientation. Complex deletions and duplication rearrangements may be the result of replication fork collapse.^29^

In addition, captured long-read sequencing significantly improves the detection of SVs in numerous genetic diseases for repeat expansion diseases in both male and female carriers.^30^^,^^31^ In our study, all female carriers were sequenced, and SVs were accurately identified in single alleles. Unlike the short-read sequencing-based method, which identifies duplications or deletions (copy number variations) using read-depth information, the long-read-based method takes advantage of split-read mapping. Hence, it can more accurately identify copy number variations in the heterozygous state. Therefore, captured long-read sequencing is a considerably more appropriate molecular diagnostic method than short-read sequencing for female carriers.

Both DMD and BMD are primarily caused by *DMD* mutations that lead to *DMD* genes of abnormal size, quantity, or function.^32^ However, occasionally, muscle biopsies may be necessary to accurately identify cases that cannot be resolved through mRNA and dystrophin protein expression studies alone.^33^ Specifically, an SNV in the exon or intron of *DMD* produces a premature termination codon or pseudoexon, resulting in DMD/BMD.^34^^,^^35^ However, these mutations are generally considered when SVs are not found in clinical gene detection, which extends the time of diagnosis. Our one-step mutation detection platform will accelerate the process of *DMD* gene detection, making the diagnosis much more efficient. But, the exception is the variation occurs during transcription only.

Furthermore, in emerging treatments for patients with DMD/BMD, antisense oligonucleotide-mediated exon skipping is one of the most promising therapeutic approaches and is expected to be applicable to most mutations, including deletions, duplications, and nonsense mutations of in-frame exons.^36^ However, it is difficult to design an antisense oligonucleotide for certain duplications and complex variations when the actual sequences are unclear. Similarly, a gene-editing therapeutic strategy also requires accurate and detailed *DMD* defect information to select the appropriate candidate. Therefore, the new era of gene therapy for DMD/BMD requires more accurate mutation detection. The long-read sequencing panel used in this study offers a highly efficient and accurate approach for the molecular diagnosis of DMD/BMD. Additionally, these findings have expanded our understanding of *DMD* gene rearrangements and provided insights into the treatment of DMD/BMD.

### Limitations of the study

In this study, we explored the captured long-read sequencing method for *DMD* mutation detection. However, some rare patients with DMD/BMD could be caused by transcription variation alone. We did not investigate the possible mechanisms underlying *DMD* rearrangement and the mRNA variation, and further research in this area is essential for our future studies.

## STAR★Methods

### Key resources table

**Table:** 

REAGENT or RESOURCE	SOURCE	IDENTIFIER
**Biological samples**		

Genomic DNA.	DMD/BMD patients peripheral blood. The raw sequence data have been deposited in the Genome Sequence Archive. The accession number is HRA004622.	Peking Union Medical College Hospital (Beijing, China).

**Oligonucleotides**		

Primers.	Please see Table S5 for detail.	Sangon Biotech (Shanghai Co., Ltd.).

**Software and algorithms**		

Guppy (high acc)	https://pypistats.org/packages/guppy3	https://pypistats.org/packages/guppy3
Nanoplexer (0.1)	https://anaconda.org/bioconda/nanoplexer/files	https://anaconda.org/bioconda/nanoplexer/files
minimap2 (2.17-r941)	https://github.com/lh3/minimap2/releases	https://github.com/lh3/minimap2/releases
mosdepth (0.2.9)	https://github.com/brentp/mosdepth/releases	https://github.com/brentp/mosdepth/releases
sniffles (1.0.12)	https://github.com/fritzsedlazeck/Sniffles	https://github.com/fritzsedlazeck/Sniffles
pepper_deepvariant (0.10.0)	https://github.com/kishwarshafin/pepper	https://github.com/kishwarshafin/pepper
clair (2.1.0)	https://github.com/quay/clair	https://github.com/quay/clair
Annovar (2017-07-17 01:17:05–0400)	https://annovar.openbioinformatics.org/en/latest/	https://annovar.openbioinformatics.org/en/latest/
AnnotSV(version 2.1)	https://github.com/lgmgeo/AnnotSV	https://github.com/lgmgeo/AnnotSV
spliceAI (1.3)	https://github.com/Illumina/SpliceAI	https://github.com/Illumina/SpliceAI
ccs (v4.2.0-1-g450908e4)	https://github.com/PacificBiosciences/ccs	https://github.com/PacificBiosciences/ccs
lima (v1.11.0-1-gec618c9)	https://lima1.readthedocs.io/en/latest/	https://lima1.readthedocs.io/en/latest/
bam2fastq (1.3.0)	https://github.com/jts/bam2fastq	https://github.com/jts/bam2fastq
deepvariant (0.10.0)	https://github.com/google/deepvariant	https://github.com/google/deepvariant

### Resource availability

#### Lead contact

Further information and requests for resources and reagents should be directed to and will be fulfilled by the lead contact, Yi Dai (pumchdy@pumch.cn).

#### Materials availability

This study did not generate new unique reagents.

#### Data and code availability

• The raw sequence data have been deposited in the Genome Sequence Archive (Genomics, Proteomics & Bioinformatics 2021) at the National Genomics Data Center (Nucleic Acids Res 2022), China National Center for Bioinformation/Beijing Institute of Genomics, Chinese Academy of Sciences. and are publicly available as of the date of publication.^37^^,^^38^ Accession numbers are listed in the key resources table.
• This paper does not report original code.
• Any additional information required to reanalyze the data reported in this paper is available from the lead contact upon request.

### Experimental model and study participant details

#### Ethics declaration

This study was approved by the Ethics Committee of Peking Union Medical College Hospital in Beijing, China. Written informed consent was obtained from all participants.

#### Human participants

A total of 129 Han Chinese individuals were included in this study. Among them, there were 100 males: 76 diagnosed with DMD with an average age of 6.55 years, 16 with BMD and an average age of 14.0 years, and seven with IMD and an average age of 10.29 years. Additionally, there was one normal subject aged 61.0 years. Furthermore, there were also 29 females: 24 asymptomatic carriers with an average age of 34.58 years, three normal subjects with an average age of 10.0 years, and two symptomatic carriers with an average age of 3.50 years. All of the subjects admitted to the Peking Union Medical College Hospital (Beijing, China), were enrolled for a single-blind, in-depth investigation and validation of *DMD* mutations using the long-read sequencing method Table S1. This study was approved by the Ethics Committee of Peking Union Medical College Hospital. Written informed consent was obtained from each participant, and all participants were anonymised for DNA sequencing and data analysis.

### Method details

#### *DMD* gene panel design

We designed a set of DNA probes with a length of 100 bp to cover the entire *DMD* gene, including all exons and introns, and the 20 kb upstream and downstream regions (GRCh37/hg19 chrX: 31115345–33377726). Probes corresponding to repetitive sequences in the human genome were excluded from the analysis.

#### DNA isolation and assessment

Genomic DNA was extracted from the peripheral blood using a genomic DNA extraction kit (Sangon Bioengineer Co., Shanghai, China) according to the manufacturer’s protocol. The quantity and quality of DNA were assessed using Qubit 3.0 (Thermo Fisher Scientific Inc., Carlsbad, CA, USA) and agarose gel electrophoresis, respectively.

#### Library preparation and sequencing

Sequencing libraries were constructed as follows: 3 μg of genomic DNA per sample was sheared to 1–6 kb fragments by g-Tube (Cat. No. 520079, Covaris, Woburn, MA) centrifugation (1,5000 × *g*, 2 min, twice). DNA was purified after fragment size characterization, after which end repair, A-tailing at the 3′ ends, and adapter ligation were performed through pre-capture amplification. After performing end repair using the NEBNext Ultra II End Repair/dA-Tailing Module (New England Biolabs, MA, USA), the adapter was attached using a NEBNext Ultra II Ligation Module (New England Biolabs). Pre-capture amplification was carried out using PrimeSTAR GXL DNA Polymerase (Takara Bio, Shiga, Japan). The PCR cycling conditions consisted of an initial denaturation at 98°C for 1 min, followed by 4 cycles of amplification (denaturation 98°C for 10 s, annealing 65°C for 15 s, and extension 68°C for 5 min). Targeted sequence capture was conducted by pooling indexed PCR products and hybridization with custom capture probes. Briefly, 1 μg of pre-capture library DNA was hybridized with solution probes and blockers (custom, IDT), along with 5 μg human Cot-1 DNA (#15279011, Thermo Fisher Scientific), at 65°C for 14 h. Biotinylated probes were captured using M-270 Streptavidin beads (#65306, Thermo Fisher Scientific). Hybridization and washing were performed according to the protocols recommended by Boke Biotechnologies. Captured fragments were amplified using PrimeSTAR GXL DNA Polymerase in a 50 μL reaction volume. The cycling conditions were as follows: initial denaturation at 98°C for 1 min, followed by 18 cycles of amplification (denaturation 98°C for 10 s, annealing 65°C for 15 s, and extension 68°C for 6 min) and a final extension at 68°C for 5 min. Finally, the PCR product was size selected using a 0.4× volume of AmpureXP beads (Beckman Coulter, USA). Purified DNA fragments were amplified by PCR, quantified, and subjected to sequencing on the long-read sequencing platforms of Oxford Nanopore PromethION using an R9.4.1 flow cell and Pacific Biosciences Sequel II, according to the manufacturer’s standard protocols (Figure 1).

#### Sequencing data analysis

For the ONT sequencing data, Guppy (version 3.0.5 + 45c3543) was used to perform base-calling in high-accuracy mode, and FASTQ reads were generated from the raw electrical signals in FAST5 format. Reads with a quality >7 were retained and demultiplexed into their corresponding samples using a Nanoplexer (version 0.1). For PB sequencing data, HiFi reads with more than five subreads were kept in the Circular Consensus Sequencing step using ccs (version v4.2.0-1-g450908e4) and further demultiplexed using lima (version v1.11.0-1-gec618c9). The long reads were then mapped to the reference genome hg19 using minimap2 (version 2.15-r906-dirty or 2.17-r941). Next, Sniffles (version 1.0.12) was used for SV calling, and Pepper_deepvariant (version 0.10.0) and deepvariant (0.10.0) were used for SNV calling for the ONT and PB platforms, respectively. SpliceAI was used for novel splice-site prediction. Consensus sequences of SV reads were generated using spoa (version v4.0.5), and insertion bases between breakpoints were manually checked using UCSC BLAT (https://genome.ucsc.edu/cgi-bin/hgBlat) (Table S4; Figure S3). Finally, the SVs in each sample were annotated with AnnotSV (version 2.1), while the SNVs and splicing events were annotated using Annovar (2017-07-17 01:17:05–0400) and spliceAI (1.3), respectively. These annotations were performed for downstream analysis.

#### Sanger sequencing

Breakpoint validation was performed using Sanger sequencing. Using Primer3web (version 4.0.0) software, the forward primer was designed using the upstream sequence of the 5′ breakpoint, and the reverse primer was designed using the downstream sequence of the 3' breakpoint (Table S5). Primers were synthesized by Sangon Biotech (Shanghai Co., Ltd.) The PCR reaction was performed with KOD FX *Neo* polymerase (TOYOBO, Osaka, Japan) using a three-step PCR protocol in a 50 μL reaction volume. The reaction mixture contained 25 μL of 2× PCR buffer, 0.4 mM of each dNTP, 0.15 μM of each primer, 1 U of polymerase, and 100 ng of genomic DNA. The PCR amplification consisted of an initial denaturation step at 94°C for 2 min, followed by denaturation at 98°C for 10 s, annealing at 60°C for 30 s, extension at 72°C for 2 min for 35 cycles, and a final elongation step of 10 min at 68°C. The presence of the expected products was confirmed by 1% agarose gel electrophoresis. The amplified products were subsequently purified using a TIANgel midi purification kit (Tiangen, Beijing) and sequenced at the Sangon Biotech facility (Shanghai, China).

### Quantification and statistical analysis

No statistical analysis was involved in this study.

### Additional resource

There are no other resources to declare.

## Supplemental information

Document S1. Figures S1‒S3

Table S1. Profile of the 129 subjects, related to Table 1, Table 2 and Figure 3

Table S2. Pathogenic mutation results of the 129 subjects across different platforms The variants were described in accordance with HGVS nomenclature and verified using the Mutalyzer program (version: 3.0.5; https://mutalyzer.nl/), related to Table 1, Table 2, Figure 1, Figure 2 and Figure 3.

Table S3. Random base insertion statistics in SV breakpoints, related to Table 1

Table S4. Software used for sequencing data analysis, related to Figure 3

Table S5. Primers used for breakpoints validation, related to Table 2
