# Supplementary material for: Uncovering the true features of dystrophin gene rearrangement and improving the molecular diagnosis of Duchenne and Becker muscular dystrophies
Source: iScience. 2023 Oct 30;26(12):108365. doi: 10.1016/j.isci.2023.108365 (PMC10690541; doi:10.1016/j.isci.2023.108365)

iScience, Volume *26*

Supplemental information

Uncovering the true features of dystrophin gene rearrangement and improving the molecular diagnosis of Duchenne and Becker muscular dystrophies

Chao Ling, Yi Dai, Chang Geng, Shirang Pan, Weipeng Quan, Qingyun Ding, Xunzhe Yang, Dongchao Shen, Qing Tao, Jingjing Li, Jia Li, Yinbing Wang, Shan Jiang, Yang Wang, Lin Chen, Liying Cui, and Depeng Wang

Figure S1. Deep intronic mutation induced pseudoexons. Long-read sequencing detected deletion and insertion in intron 48 of subject D116, which resulted in a 161 bp pseudoexon suggested by muscle biopsy RNA-Seq. Related to Table 1.

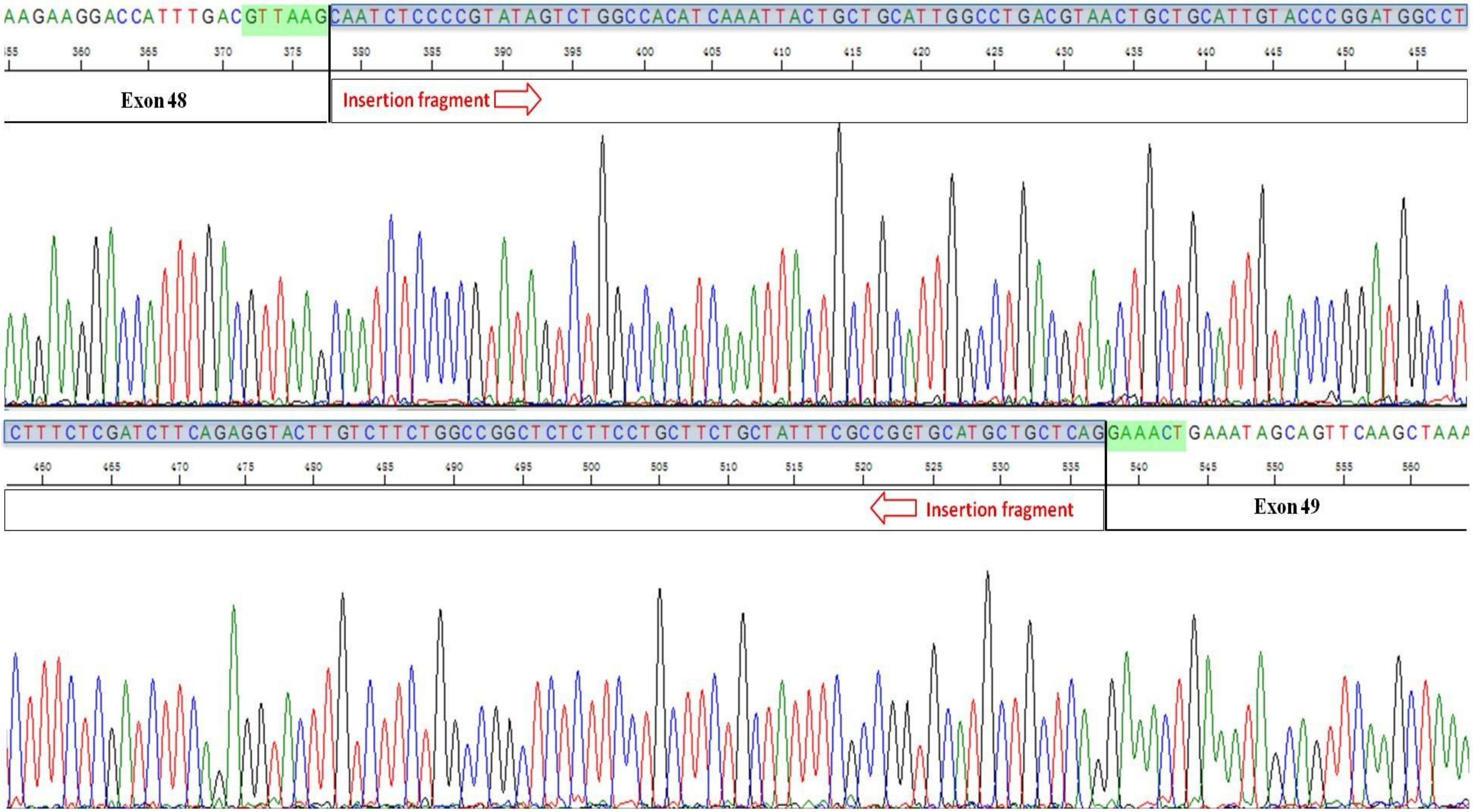

Figure S2. Inversion and translocation verification with Sanger sequencing. All rearrangements were verified by sequencing the junction region. The orange box represents the breakpoints, and the blue box represents the normal sequence location. A shaded sequence represents an insertion sequence. Related to Table 2.

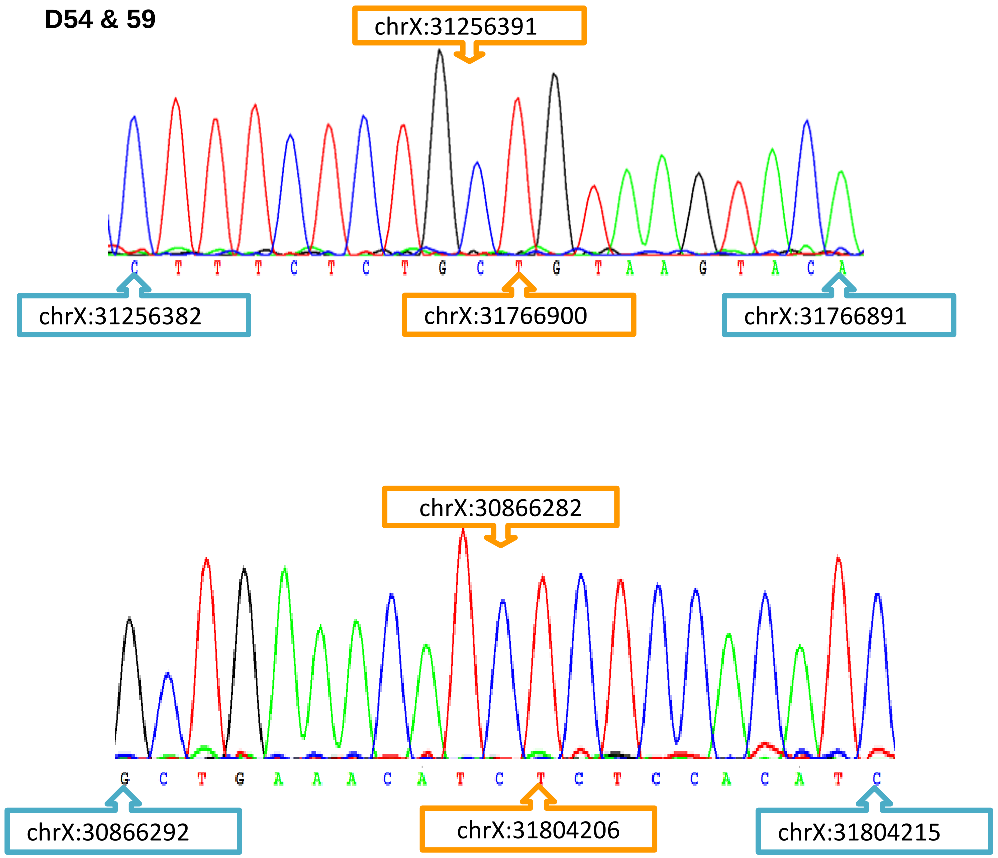

D78

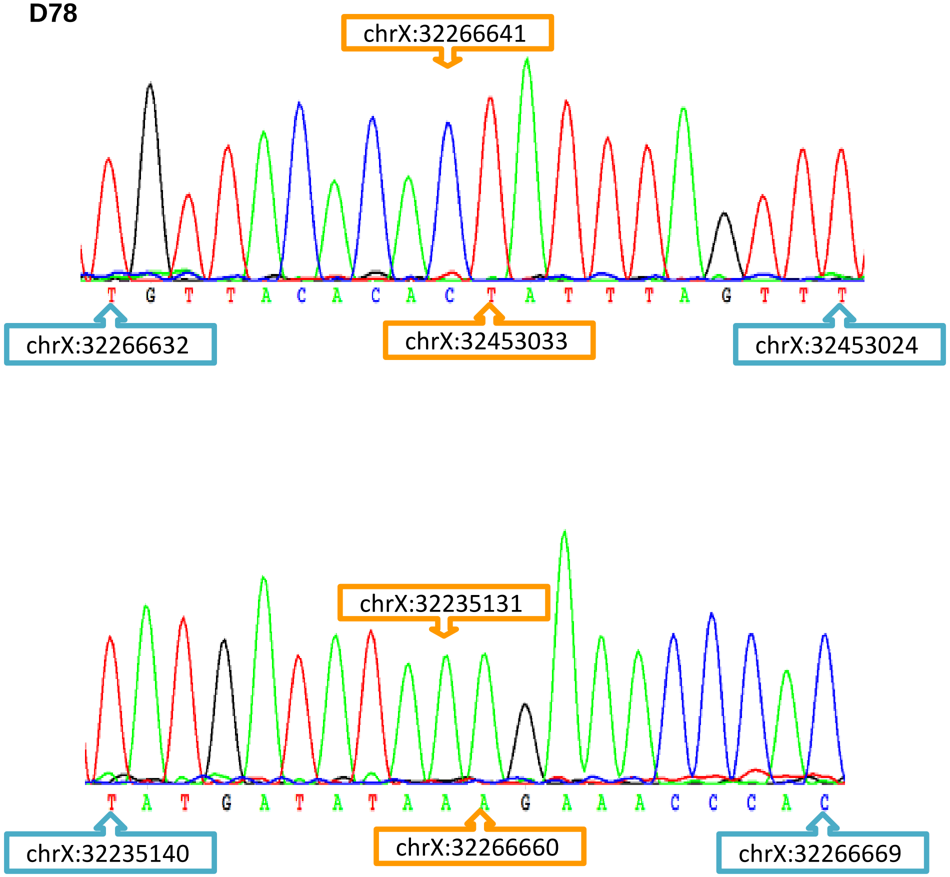
chrX:32266641
T G T T A C A C A C T A T T T A G T T T
chrX:32266632
chrX:32453033
chrX:32453024
chrX:32235131
T A T G A T A T A A A G A A A C C C A C
chrX:32235140
chrX:32266660
chrX:32266669

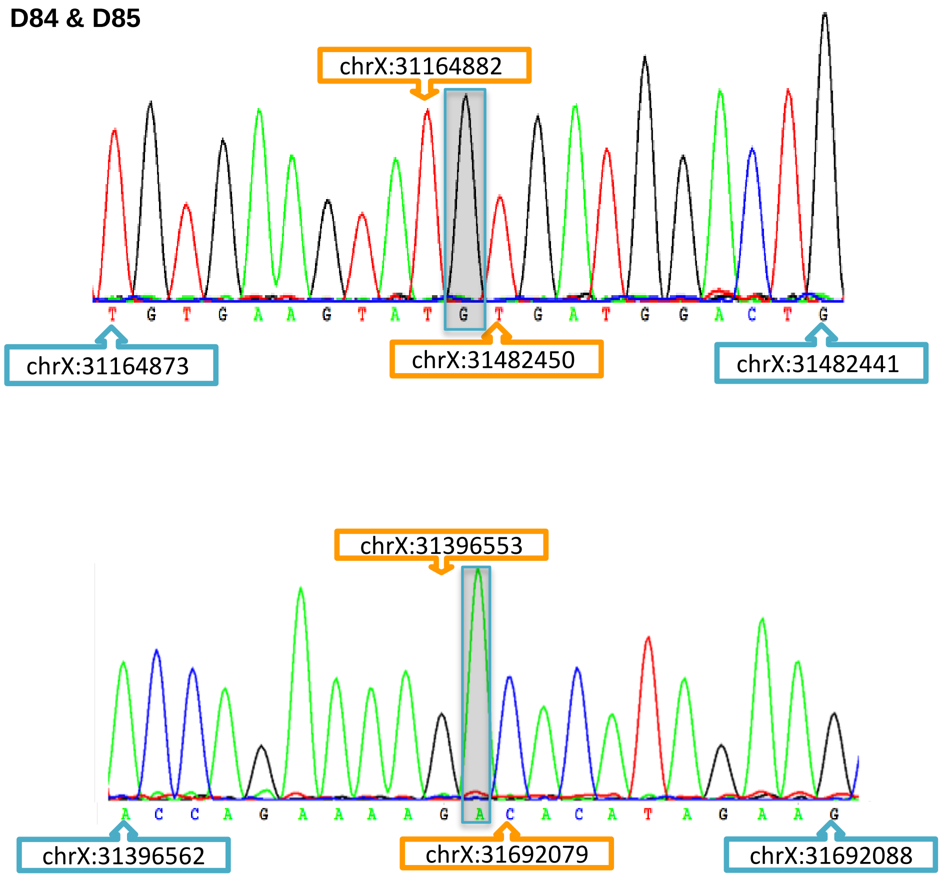
D84 & D85
chrX:31164882
T G T G A A G T A T G T G A T G G A C T G
chrX:31164873
chrX:31482450
chrX:31482441
chrX:31396553
A C C A G A A A A G A C A C A T A G A A G
chrX:31396562
chrX:31692079
chrX:31692088

D89 & D90

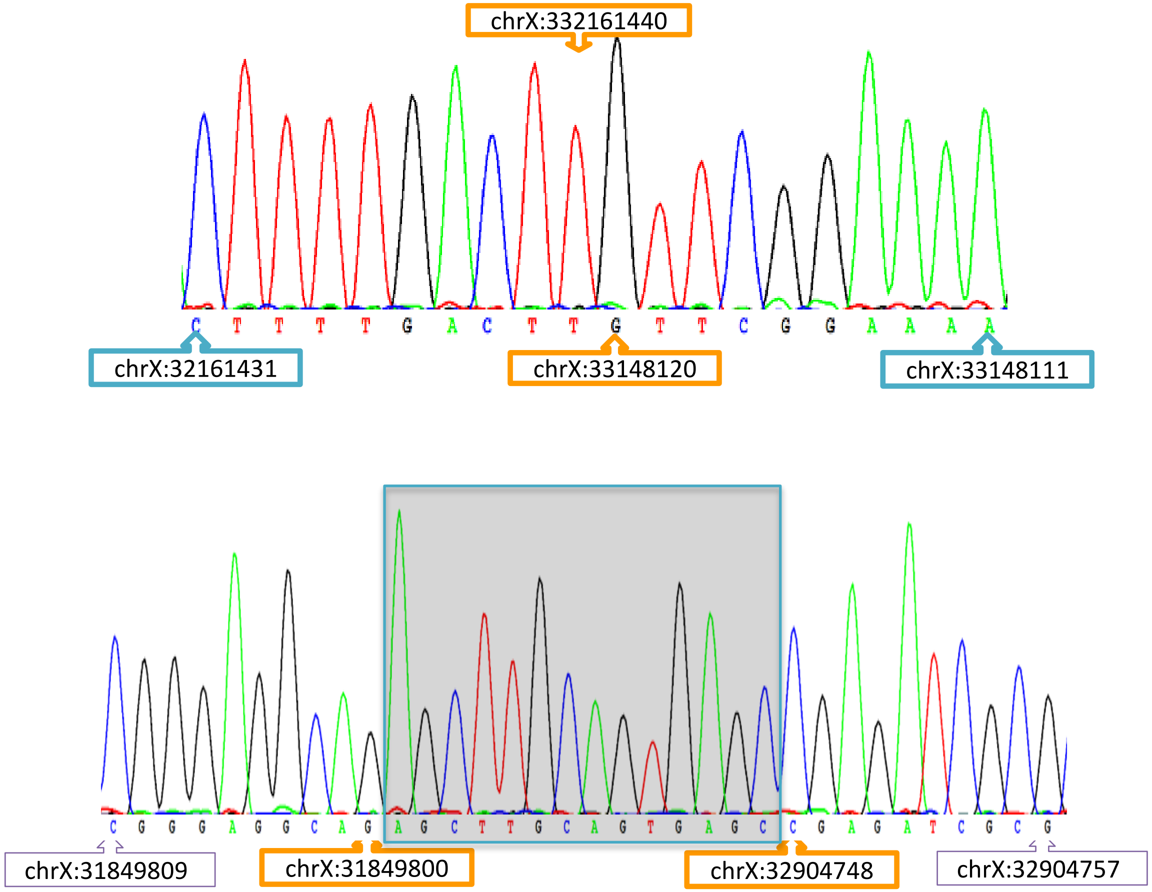
chrX:332161440
chrX:32161431
chrX:33148120
chrX:33148111
chrX:31849809
chrX:31849800
chrX:32904748
chrX:32904757

D133

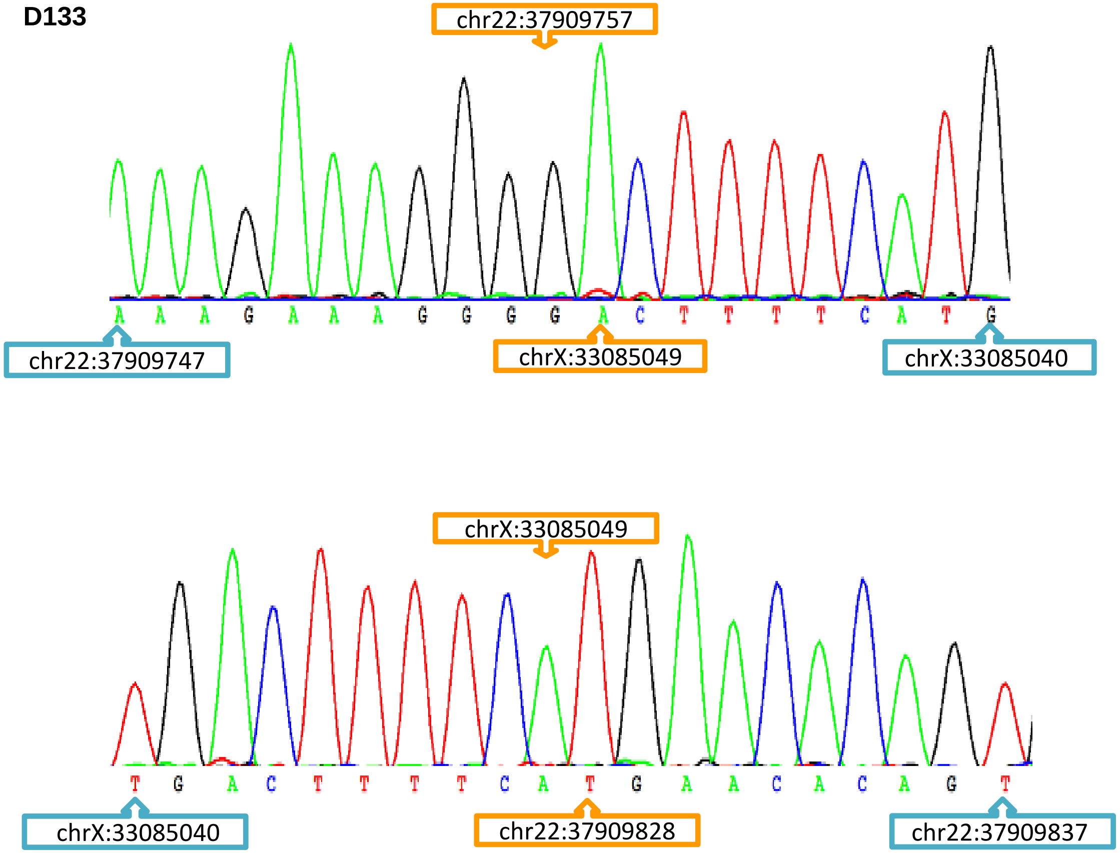

chr22:37909757
AAAGAAAGGGGACTTTTCATG
chr22:37909747
chrX:33085049
chrX:33085040
chrX:33085049
TGACTTTTCATGAACACAGT
chrX:33085040
chr22:37909828
chr22:37909837

D134

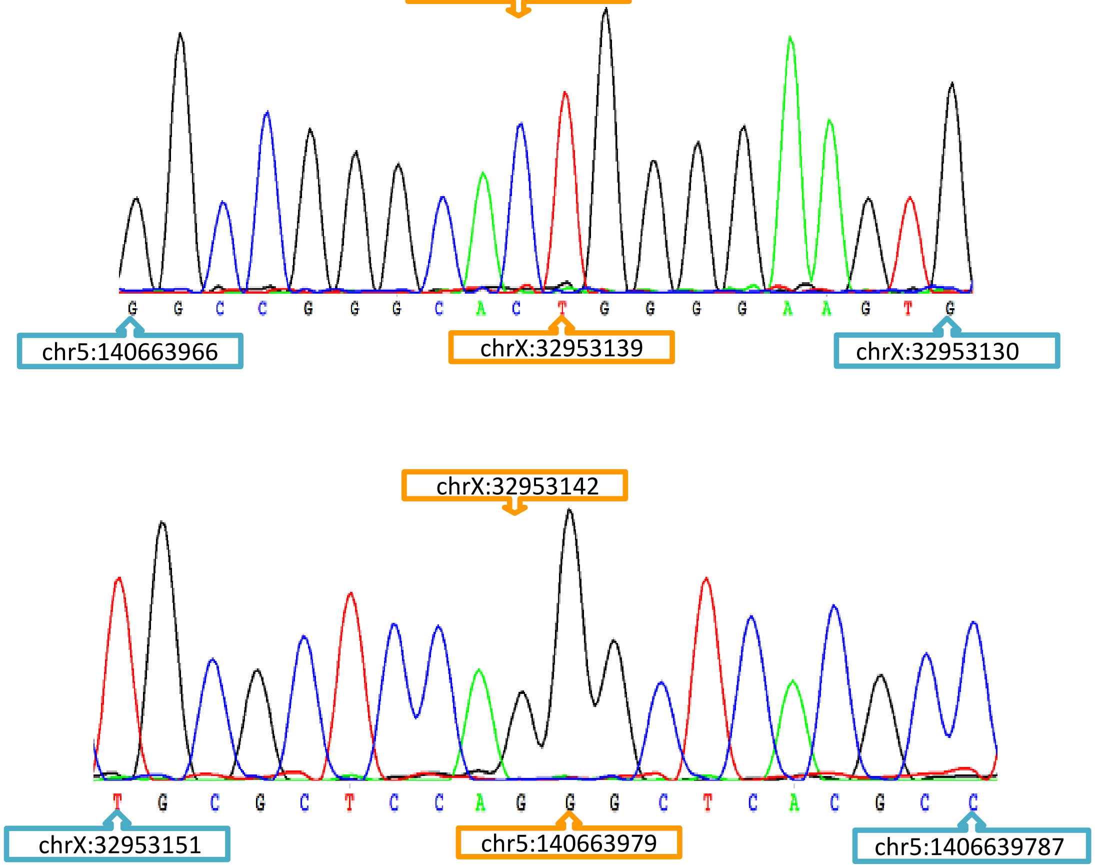

chr5:140663975
G G C C G G G C A C T G G G G A A G T G
chr5:140663966
chrX:32953139
chrX:32953130
chrX:32953142
T G C G C T C C A G G G C T C A C G C C
chrX:32953151
chr5:140663979
chr5:1406639787

D135

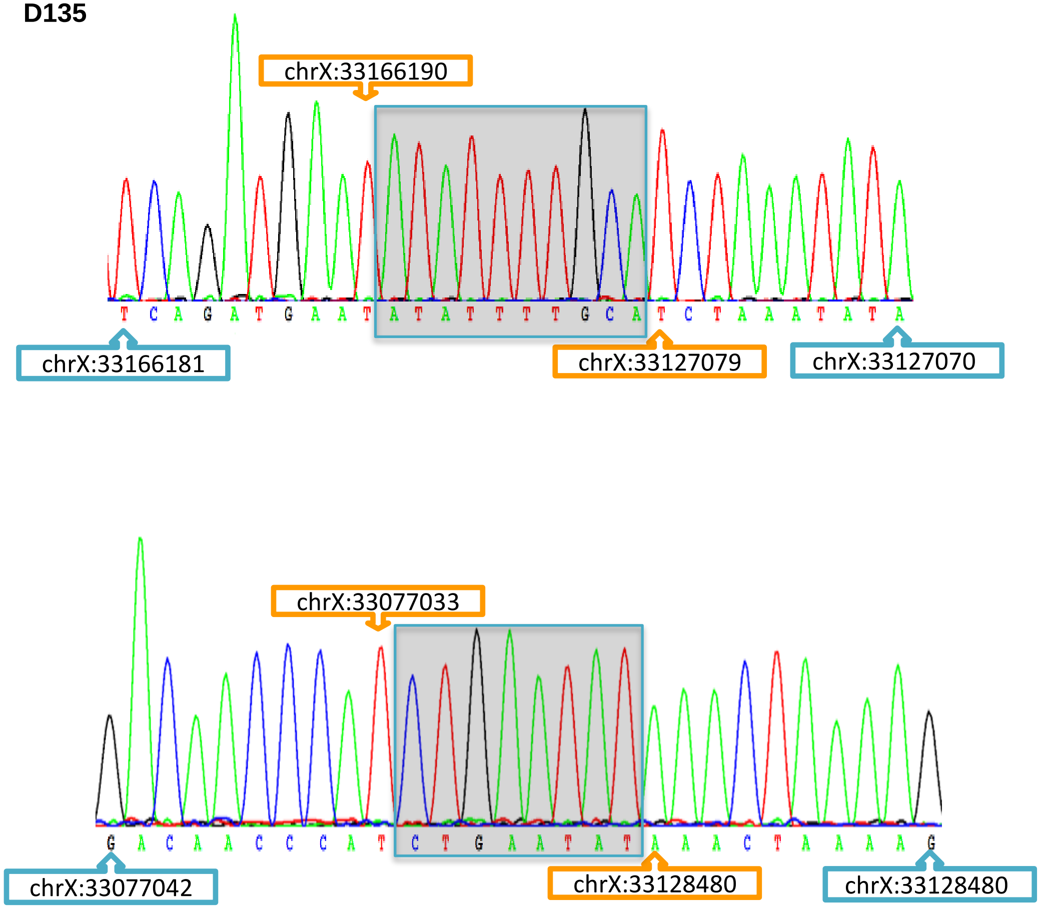
chrX:33166190
TCAGATGAATATATTTTGCATCTAAATATA
chrX:33166181
chrX:33127079
chrX:33127070
chrX:33077033
GACAACCCATCTGAATATAAACTAAAAG
chrX:33077042
chrX:33128480
chrX:33128480

Figure S3. Flow chart of long-read sequencing data analysis. Data generated from PB and ONT platforms were combined for subsequent processing and analysis. Related to Figure 3.

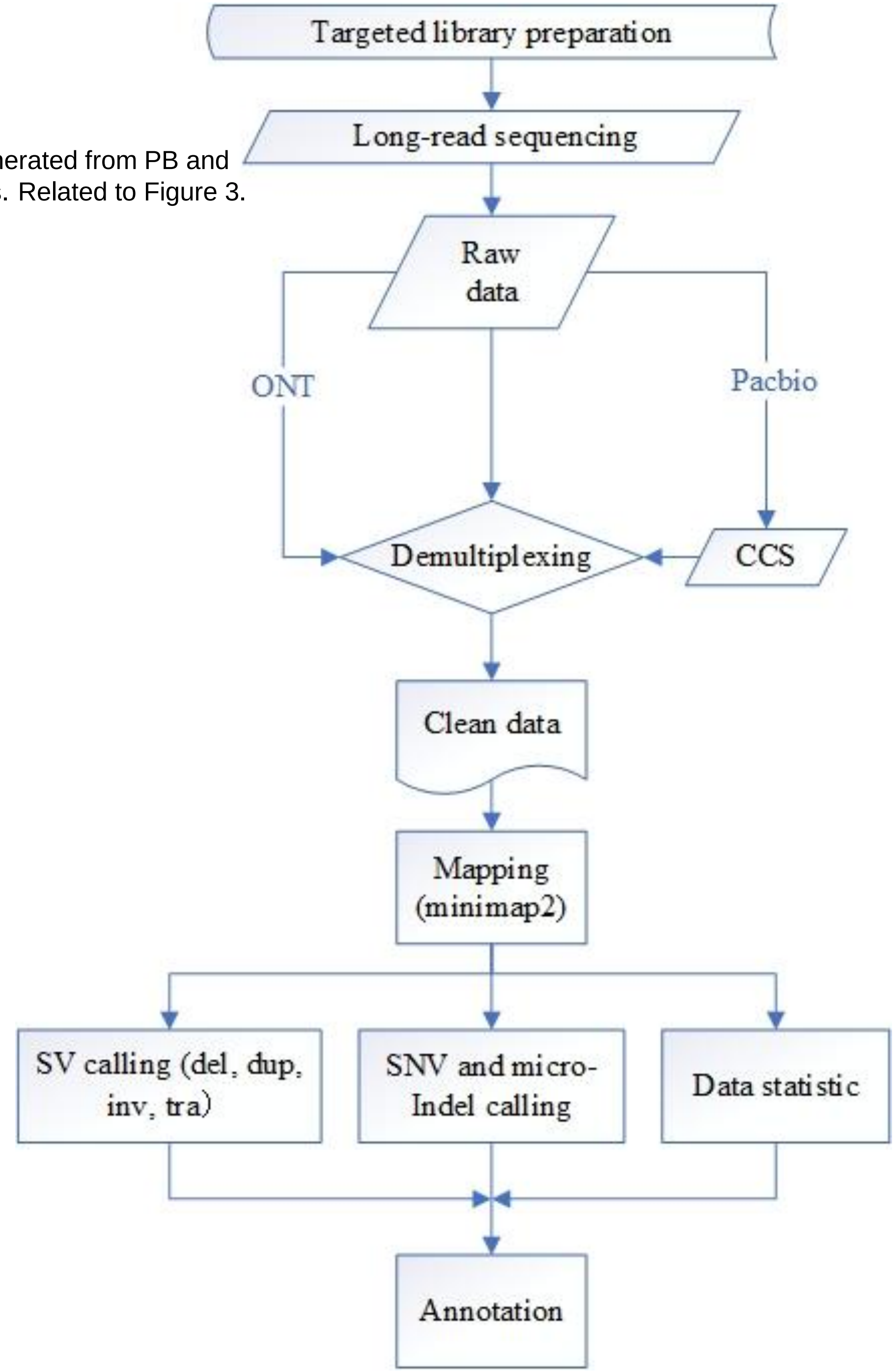

Supplement: Document S1. Figures S1‒S3 [file mmc1.pdf]
